# Down-regulated circPAPPA suppresses the proliferation and invasion of trophoblast cells via the miR-384/STAT3 pathway

**DOI:** 10.1042/BSR20191965

**Published:** 2019-09-06

**Authors:** Wenbo Zhou, Huiyan Wang, Jingjing Yang, Wei Long, Bin Zhang, Jianbing Liu, Bin Yu

**Affiliations:** Changzhou Maternity and Child Health Care Hospital affiliated to Nanjing Medical University, Changzhou 213003, Jiangsu, China

**Keywords:** circRNA, hsa_circ_0088227, miR-384, Preeclampsia, Stat3

## Abstract

Preeclampsia (PE) is the main cause of maternal death in primipara, and commonly results in severe maternal and neonatal complications such as multiple organ dysfunction syndrome. However, the exact pathogenesis of this disease remains unclear. Circular RNAs (circRNAs) are noncoding RNAs that have been shown to be extensively involved in numerous physiological processes, but there is limited knowledge of their functions and mechanisms in PE. In the present study, we found the expression of a circRNA, hsa_circ_0088227 (circRNA of pregnancy-associated plasma protein A, circPAPPA), was down-regulated in both placenta and plasma samples from subjects with PE. Knockdown of circPAPPA led to decreased proliferation and invasion in HTR8-S/Vneo trophoblast cells. miR-384 was identified as a direct target of circPAPPA, and the gene encoding signal transducer and activator of transcription 3 (STAT3) was targeted by miR-384. We found that miR-384 was unregulated in PE, and overexpression of miR-384 could inhibit cell proliferation and invasion. In addition, we showed that the expression of STAT3 was decreased with knockdown of circPAPPA or the overexpression of miR-384 in trophoblast cells, but this decrease was partially reversed when co-transfection was performed with mimics of miR-384 inhibitor and si-circPAPPA. Together, these results suggest that down-regulation of circPAPPA facilitates the onset and development of PE by suppressing trophoblast cells, with involvement of the miR-384/STAT3 signaling pathway. Our study significantly increases the understanding of the occurrence and development of PE, and also provides a molecular target for the treatment of this disorder.

## Introduction

Preeclampsia (PE) is a disorder occurring during pregnancy that is characterized by hypertension, proteinuria, edema, and progressive damage of cardiac and renal functions in pregnant women after 20 weeks of pregnancy [1,2]. The global incidence of PE is approximately 5–8%, and it has been documented as one of the major causes of maternal and perinatal deaths [3]. The etiology and pathogenesis of PE is unclear, but the placenta has been found to be closely related to the pathogenesis of this disorder [4,5].

Circular RNA (circRNA) is a ring-like RNA molecule that is produced by reverse splicing of precursor RNA after transcription. It is involved in gene transcription and post-transcriptional regulation. Using transcriptome and bioinformatics analyses, the rapid development of molecular biology and high-throughput sequencing technology have facilitated increased knowledge of the mechanisms of action of circRNAs in various disorders. CircRNAs have been reported to play important regulatory roles in tumors, diabetes mellitus, and neurological diseases [6–8]. In pregnancy-related diseases, circRNA has been reported to be associated with gestational diabetes mellitus [9], and differential expression profiles of circRNAs in the plasma and placenta of pregnant women with PE have been observed [10,11]. These results suggest that circRNAs might be associated with PE and may play a regulatory role. However, how these differentially expressed circRNAs affect the function of trophoblasts and thus participate in the development of PE, has not yet been reported.

Previously, we identified differentially expressed circRNAs by RNA sequencing and verified their potential functions using qPCR and bioinformatics [12]. In particular, circRNA of pregnancy-associated plasma protein A (circPAPPA) (hsa_circ_0088227), which originates from the pregnancy-associated plasma protein A (PAPPA) gene, attracted our attention. In the present study, we aimed to characterize its potential function and mechanism, which might be involved in the occurrence and development of PE. After transfection of an siRNA of circPAPPA into HTR8-S/Vneo cells, the effects on cell proliferation and invasion were observed, and potential molecular mechanisms were identified.

## Materials and methods

### Sample collection

From August 2017 to October 2018, 83 pregnant women who underwent a cesarean section at the Changzhou Maternity and Child Health Care Hospital were recruited for the present study, including 40 women diagnosed with early-onset PE and 43 normal pregnant women. The clinical characteristics of the normal and PE groups are shown in Table 1. Pregnant women who had primary hypertension, multiple pregnancies, chromosomal abnormalities, congenital malformations, or suspected perinatal infection were excluded from the study. After signing an informed consent form, plasma samples were collected from them before the cesarean section. The placental tissues were obtained within 10 min after the cesarean section, cut into 1 g pieces, washed with cold saline, and then cut into smaller pieces and frozen at −80°C immediately after adding 1 ml TRIzol reagent for RNA extraction.

**Table 1:** Clinical characteristics of normal and PE group

	Control (*n*=43)	PE (*n*=40)	*P*-value
Age (years)	30.4 ± 2.2	30.11 ± 2.77	0.61
Urine protein (g/24 h)	0.06 ± 0.07	3.58 ± 1.54	<0.01
Gestational weeks	38.45 ± 1.13	36.2 ± 0.91	<0.01
Systolic blood pressure (mmHg)	117.4 ± 6.74	161.9 ± 6.43	<0.01
Diastolic blood pressure (mmHg)	77.65 ± 7.1	104.74 ± 13.52	<0.01
Body mass index (kg/m^2^)	24.37 ± 1.55	24.71 ± 1.58	0.32
Primiparous percentage (%)	62.5	60.4	>0.05
Smoking	4	3	>0.05
Neonatal weight(g)	3383 ± 299.5	2557.5 ± 413.5	<0.01

### Quantitative real-time polymerase chain reaction assay

Total RNA was extracted using TRIzol reagent (Thermo Fisher Scientific, Waltham, MA, U.S.A.). After treatment with RNase R, the total RNAs were divided into two groups: the RNase R+ and RNase R− groups. cDNA was obtained using a reverse-transcription kit (MM-MLV; Promega, Madison, WI, U.S.A.), and RT-PCR was performed using primer with the sequences listed in Table 2. PCRs were conducted as follows: denaturation at 95°C for 10 min, amplification over 40 cycles of 95°C for 10 s and 60°C for 15 s, followed by treatment at 72°C for 30 s. The relative expression levels of target genes were determined by comparing the *C*_t_ values of the target genes to those of the glyceraldehyde 3-phosphate dehydrogenase (GAPDH) or U6 genes. The 2^−ΔΔ*C*^_t_ method was used to determine the relative gene expression levels [13].

**Table 2:** Primer sequences used in the present study

ID	Primer sequences
circPAPPA	forward 5′-TGTGGCCTCCATCCTACATCTCAA-3′
	reverse 5′-CAGGTGCGGACACTGGACTTAC-3′
PAPPA	forward 5′-TCAGGCGCAGCAACTCTTTC-3′
	reverse 5′-AGCCCAGGACACTGTTGAAT-3′
STAT3	forward 5′-CTCAAGATTGACCTAGAGAC-3′
	reverse 5′-TGGGATTGTTGGTCAGCATG-3′
miR-384	forward 5′-TGTTAAATCAGGAATTTTAA-3′
	reverse 5′-TGTTACAGGCATTATGAA-3′
U6	forward 5′-TGCGGGTGCTCGCTTCGCAGC-3′
	reverse 5′-CCAGTGCAGGGTCCGAGGT-3′
GAPDH	forward 5′-TGACTTCAACAGCGACACCCA-3′
	reverse 5′-CACCCTGTTGCTGTAGCCAAA-3′

### Cell culture and transfection

HTR-8/SVneo cells were obtained from the Chinese Academy of Sciences Committee (Shanghai, China) and cultured in RPMI 1640 medium containing 10% fetal bovine serum (FBS) (Gibco, Scotts Valley, CA, U.S.A.) at 37°C, in a 5% CO_2_ incubator. For the transfection of siRNAs, cells (2 × 10^5^) were seeded into six-well plates. After 24 h, they were transfected with 100 nM siRNA using Lipofectamine 3000 (Invitrogen, Carlsbad, CA, U.S.A.). The sequence of the siRNA for circPAPPA (si-circPAPPA) was 5′-GAAAUUCGUAGACAUGUCAUCdTdT-3′; that for the negative control siRNAs (si-NC) was 5′-UUCUCCGACGUGUCACGUdTdT-3′; that for the miR-384 mimic was 5′-AUUCCUAGAAAUUGUUCAUA-3′; that for the NC mimic was 5′-CAGUACUUUGUUAGUACAA-3′; and that for the miR-384 inhibitor was 5′-UAUGAACAAUUUCUAGGAAU-3′. All sequences were synthesized by Ribo (Guangzhou, China).

### Cell invasion assay

The *in-vitro* Transwell assay (Corning, NY, U.S.A.) was used to evaluate the invasion capacity of the trophoblast cells. The upper chamber of the Transwell chamber was coated with Matrigel gel (BD Biosciences, San Jose, CA, U.S.A.) or left non-coated. HTR-8/SVneo cells were cultured in serum-free medium for 24 h. The cell density was adjusted to 1 × 10^5^ cells/ml, 100 μl of cell suspension was added to the top chamber, and 500 μl of culture medium containing 20% FBS was added to the lower chamber. After incubation at 37°C with 5% CO_2_ for 48 h for the invasion assay, the cells and the Matrigel on the top surface of the Transwell membrane were removed with a cotton swab. The cells on the bottom surface of the membrane were fixed with cold paraformaldehyde and stained with 500 μl of 0.1% Crystal Violet (Sigma–Aldrich, St. Louis, MO, U.S.A.). Five fields on each membrane were randomly selected, and the invaded cells were counted.

### CCK8 assay

The CCK8 assay (Beyotime, China) was performed to assess cell proliferation. The cells were adjusted to 2 × 10^5^ cells/well, seeded into a 96-well plate at 24 h after transfection, and cultured for different times (24, 48, and 72 h). Then, 10 μl CCK8 solution was added to each well, followed by incubation for 1.5 h at 37°C. The optical density value at 450 nm was measured using a SpectraMax M5 ELISA plate reader (BioTek, Winooski, VT, U.S.A.).

### 5-ethynyl-2′-deoxyuridine assay

A total of 4 × 10^3^ cells per well were seeded into 96-well plates and cultured overnight, and then washed with phosphate-buffered saline (PBS), fixed with 4% paraformaldehyde for 30 min, and incubated with 2 mg/ml glycine for 5 min. Based on the kFluor488-EdU (5-ethynyl-2′-deoxyuridine) manufacturer’s instructions (Keygentec, Nanjing, China), 200 μl of 1× Apollo dyeing solution was added to each well, followed by incubation at room temperature for 30 min. Next, 100 μl of 0.5% Triton X-100 was used to wash the cells two- to three-times (10 min per wash). Following staining with Hoechst 33342 at room temperature for 30 min in darkness and one or two washes with PBS, the cells were observed using a Micro system (ImageXpress, Downingtown, PA, U.S.A.). Five fields were randomly selected and photographed, and the number of EdU-positive cells was calculated.

### Luciferase reporter assay

THTR8-S/Vneo cells were co-transfected with a bioluminescent reporter vector (psicheck2.0-circPAPPA-wt or mut/Stat3-wt or mutant) and the miR-384 or NC mimic using Lipofectamine 3000 (Invitrogen) according to the manufacturer’s protocol. After 48 h, the cells were harvested, and luciferase activity was measured using a Dual-Luciferase Reporter Assay Kit (TransGen, Beijing, China).

### Western blotting

Total protein was extracted from transfected cells or placenta tissues using RIPA lysis buffer containing 1 mM phenylmethylsulfonyl fluoride (Beyotime, Beijing, China), and then the protein concentration was determined using the BCA Protein Assay Kit (Beyotime). Fifty microgram protein was resolved using 10% SDS/PAGE and transferred on to polyvinylidene difluoride membranes (Sigma–Aldrich). The membranes were blocked using 5% nonfat milk in TBST at room temperature for 1 h, and then incubated overnight at 4°C with appropriately diluted primary antibodies as follows: anti-signal transducer and activator of transcription 3 (STAT3) (1:1000), anti-phospho-Stat3 (Tyr^705^, 1:1000), or anti-GAPDH (1:1000), all purchased from the Biosynthesis Biotechnology Co. (Beijing, China). After three washes with PBST, the membranes were incubated with horseradish peroxidase-labeled secondary antibodies (anti-rabbit or anti-mouse; Beyotime) for 1 h at room temperature. The membranes were then washed three times with PBST, and treated for 1 min in darkness with 1 ml of enhanced chemiluminescence reaction reagent (BeyoECL Plus, Beyotime). The membranes were then observed and photographed using a 5200 Luminescence imaging system (Tanon, Shanghai, China). Protein expression was analyzed by obtaining optical densities.

### Fluorescence *in situ* hybridization

A Cy3-labeled oligonucleotide probe for circPAPPA (Cy3-5′-AGGATGACATGTCTACGAATT-3′) and a fluorescein isothiocyanate (FITC)-labeled miR-384 probe (FITC-5′-TATGAACAATTTCTAGGAAT-3′) were used for RNA fluorescence *in situ* hybridization (FISH). After pre-hybridization at 55°C for 2 h, mounted cell sections were hybridized with the probes at 37°C overnight. After staining with 4′,6-diamidino-2-phenylindole, the slides were photographed and images were acquired using a laser confocal microscope (TCS SP2, Leica, Wetxlar, Germany).

### Statistical analysis

The data were analyzed using SPSS 22 statistical software, and expressed as the mean and standard error. The *t* test or one-way analysis of variance was used to determine statistical differences. A value of *P*<0.05 represented statistical significance.

## Results

### Characterization of circPAPPA in trophoblast cells of PE patients

**Figure 1:**
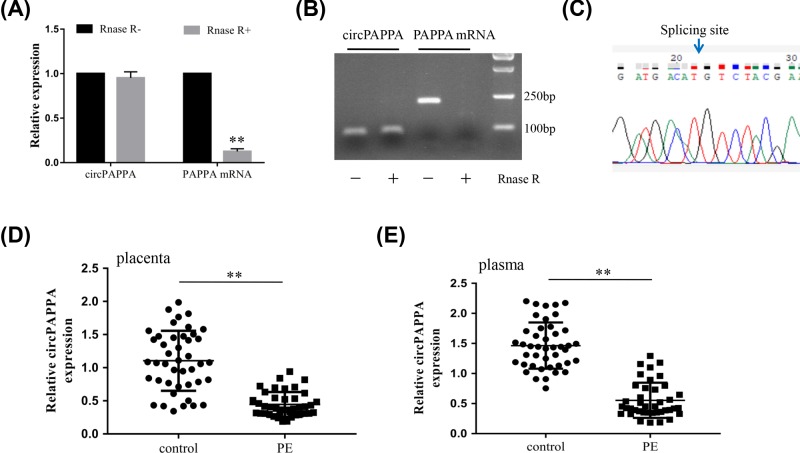
Characterization of circPAPPA in trophoblasts of PE (**A**) Quantification of circPAPPA and PAPPA mRNA in HTR8-S/Vneo cells treated with or without RNase R. (**B**) PCR products of circPAPPA and PAPPA mRNA resolved using a 1.5% agarose gel. (**C**) A divergent primer was designed to detect circPAPPA, and its back-splicing junction was validated by Sanger sequencing. (**D,E**) Relative expression levels of circPAPPA in placenta (D) and plasma (E) samples from patients with PE and normal pregnant women were determined using qRT-PCR. **P<0.01.

CircPAPPA originates from the *PAPPA* gene, which contains 22 exons encoding 1627 amino acids, and is located on chromosome 9. CircPAPPA is generated by back-splicing exon 7 of the *PAPPA* gene, whose biological function has not been determined. RNA harvested from HTR8-S/Vneo trophoblast cells, was treated with or without RNase R, and then subjected to quantitative real-time polymerase chain reaction (qRT-PCR) to check the expression of PAPPA and circPAPPA. The results showed that the expression of the PAPPA mRNA RNase R-treated group was significantly lower than that in the RNase R-free group (Figure 1A), but there was no difference between the expression of the circPAPPA RNase R and RNase R-free groups. These results indicate that the circular structure of circPAPPA was resistant to RNase R. Images of the PCR products of circPAPPA and PAPPA mRNA on 1.5% agarose gels also confirmed this conclusion (Figure 1B). The resulting RNA circle ligation site was located using Sanger sequencing (Figure 1C). The expression of circPAPPA was then measured in 40 PE patients and 43 adjacent normal pregnant women using qRT-PCR. Compared with normal pregnant women, the expression levels of circPAPPA in the placenta (Figure 1D) and plasma (Figure 1E) were all lower in the PE group.

### Down-regulation of circPAPPA inhibited proliferation and suppressed trophoblast cell invasion

**Figure 2:**
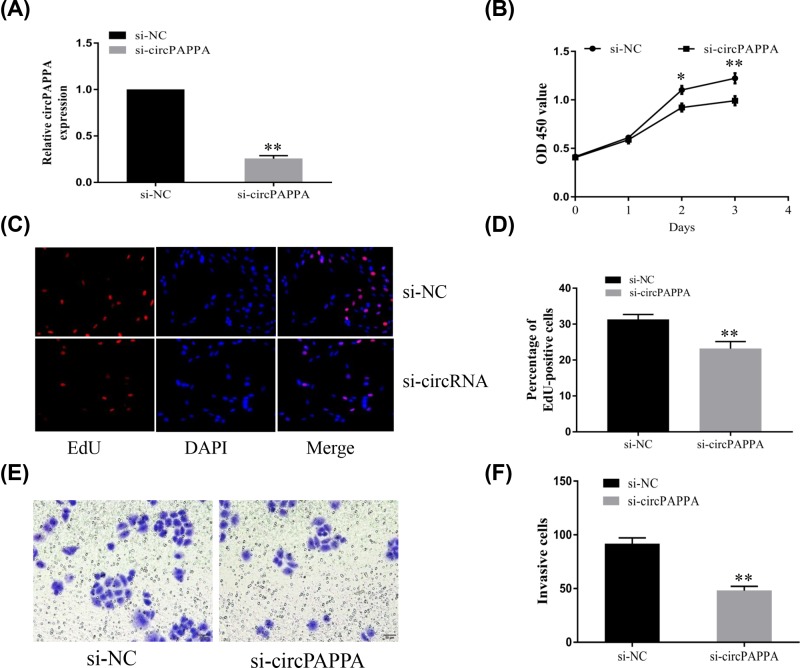
Down-regulation of circPAPPA inhibited proliferation and suppressed trophoblast cell invasion (**A**) HTR-8/SVneo cells were transfected with circPAPPA siRNA or si-NC, and circPAPPA expression was determined using qPCR. (**B**) Knockdown of circPAPPA inhibited cell proliferation, as revealed by the CCK8 assay of HTR-8/SVneo cells at different time periods after transfection. (**C,D**) Trophoblast cell proliferation was measured using the EdU assay. (**E,F**) An *in-vitro* Transwell assay was conducted to evaluate the invasion capability of HTR-8/SVneo cells after transfection with si-circPAPPA or si-NC. *P<0.05; **P<0.01.

**Figure 3:**
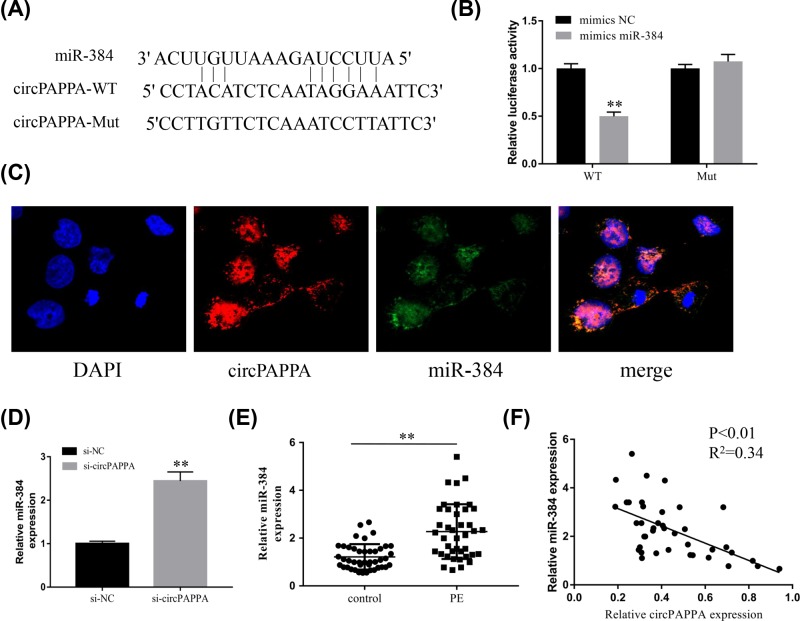
miR-384 is a direct target of circPAPPA (**A**) Predicted binding sites of miR-384 and circPAPPA (wild-type and mutated). (**B**) Luciferase reporter assays were used to determine the interaction between miR-384 and circPAPPA. (**C**) Confocal FISH was performed to determine the subcellular co-localization of circPAPPA and miR-384 in trophoblast cells. (**D**) The expression of miR-384 was measured by PCR after transfection with si-circPAPPA or si-NC. (**E**) Relative expression levels of miR-384 in placenta samples from patients with PE and normal pregnant women, determined by qPCR. (**F**) Pearson’s correlation analysis indicated an inverse correlation between the miR-384 and circPAPPA levels in placenta tissues. **P<0.01.

To investigate the biological function of circPAPPA, we knocked down its expression in HTR8-S/Vneo trophoblast cells (Figure 2). SiRNAs targeting the back-splice junction of circPAPPA (si-circPAPPA) were designed and synthesized. Transfection with si-circPAPPA showed a significant reduction in its expression (Figure 2A). The results of the CCK8 assay (Figure 3B) and the EdU assay (Figure 3C,D) suggest that both cell viability and proliferation were reduced 48 h after transfection. Accumulating evidence indicates that trophoblast cell invasion is closely related to the occurrence and development of PE [14,15]. To investigate whether circPAPPA plays a role in the regulation of trophoblast cell invasion, an *in-vitro* Transwell assay was conducted. The results showed that knockdown of circPAPPA expression suppressed the cell invasion capability in trophoblast cells (Figure 2E), and there were fewer invading cells than in the si-NC group (Figure 2F).

### miR-384 is a direct target of circPAPPA

CircRNAs are widely considered to act as miRNA sponges, helping regulate gene expression. Bioinformatics analyses (Circinteractom and TargetScan) showed that miR-384 contains binding sites for circPAPPA (Figure 3A). The dual luciferase reporter assay indicated that luciferase activity was decreased when miR-384 mimics and the wild-type form of circPAPPA were co-transfected (Figure 3B). In addition, FISH results showed that circPAPPA co-localized with miR-384 in the cytoplasm (Figure 3C). We consistently found that circPAPPA knockdown led to increased levels of miR-384 in HTR8-S/Vneo cells (Figure 3D). In addition, we found that the expression of miR-384 was also significantly increased in the placenta of PE pregnant women, compared with normal controls (Figure 3E). Pearson’s correlation analysis showed that miR-384 expression was negatively associated with the expression of circPAPPA in placental tissues (Figure 3F; *P*<0.01; *R^2^* = 0.34). Collectively, these results indicate that circPAPPA targeted miR-384 directly and could act as an miR-384 sponge.

### Overexpression of miR-384 inhibited trophoblast cell proliferation and suppressed invasion

**Figure 4:**
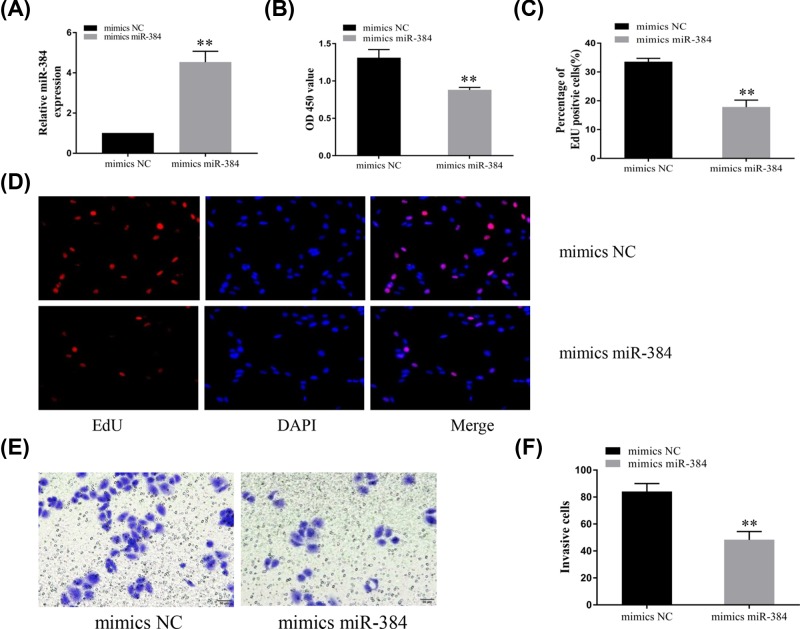
Overexpression of miR-384 inhibited trophoblast cell proliferation and suppressed invasion (**A**) HTR-8/SVneo cells were transfected with miR-384 mimics or a normal control, and miR-384 expression was determined using qPCR. (**B**) The CCK8 assay was used to detect cell viability after miR-384 overexpression. (**C,D**) Trophoblast proliferation was measured using EdU. (**E,F**) An *in-vitro* Transwell assay was conducted to evaluate the invasion capability of trophoblast cells after transfection with miR-384, NC mimics, or NC. **P<0.01.

Many studies have reported that miR-384 acts as a tumor suppressor and can inhibit cell proliferation and invasion in various tumors [16–18]. However, there has been no similar report of its involvement in PE. To validate its role in PE, we transfected miR-384 and normal control (NC) mimics into HTR8-S/Vneo trophoblast cells. The expression of miR-384 was significantly increased after transfection (Figure 4A). The CCK8 (Figure 4B) and EdU (Figure 4C,D) expression levels showed that miR-384 overexpression significantly suppressed cell proliferation. In addition, Transwell results showed that the invasive ability of trophoblast cells was decreased after overexpression of miR-384 (Figure 4E,F).

### Examination of the role of regulation of circPAPPA and miR-384 in trophoblast cell function by targeting STAT3

**Figure 5:**
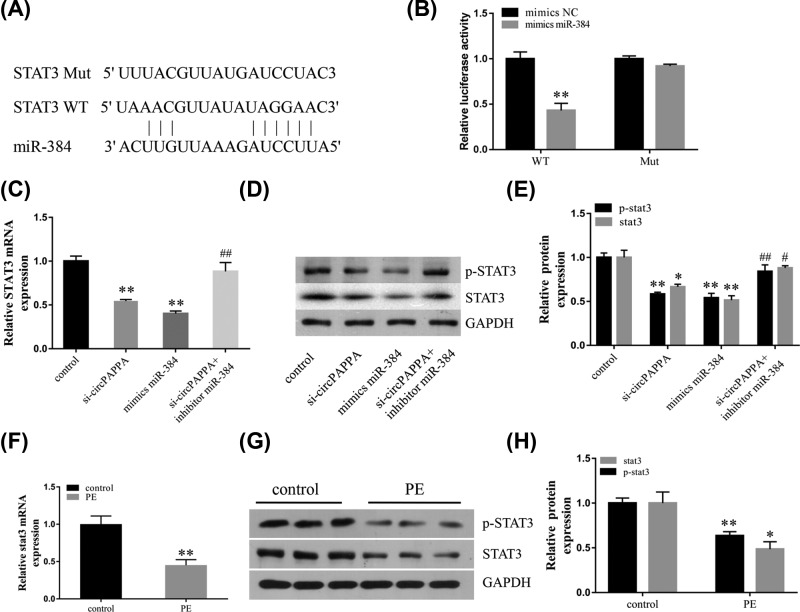
Effect of circPAPPA and miR-384 on trophoblast cell function shown by targeting STAT3 (**A**) Putative binding sites within miR-384 and circPAPPA predicted by bioinformatics analysis. (**B**) Luciferase reporter assay results showing that miR-384 overexpression reduced the STAT-wild-type luciferase activity. (**C,D**) Relative levels of STAT3 mRNA (C) and STAT3 and p-STAT3 proteins (D) after transfection with si-circPAPPA, miR-384 mimics, or miR-384 inhibitor and si-circPAPPA. (**E**) Quantification of protein expression. (**F**) Relative levels of STAT3 mRNA in placenta tissues of PE and normal controls. (**G,H**) Relative expression levels of p-STAT3 and STAT3 proteins in placenta tissues of PE and normal controls determined using Western blotting. *Compared with the control group; *P<0.05; **P<0.01; ^#^compared with the si-circPAPPA group; ^#^*P*<0.05; ^##^*P*<0.01.

MiRNAs have been shown to have a regulatory function through targeting the 3′ UTR of mRNA. We therefore predicted the target gene of miR-384 using bioinformatics analysis (TargetScan, PicTar, and miRanda). We found that the 3′ UTR of STAT3 possesses a potential binding site for miR-384 (Figure 5A). A luciferase reporter assay showed that miR-384 mimics suppressed the luciferase activity of the wild-type STAT3 reporter (Figure 5B), confirming that this gene is targeted directly. In addition, we found that STAT3 mRNA expression was decreased with knockdown of circPAPPA or overexpression of miR-384, but this decrease was partly reversed when the miR-384 inhibitor and si-circPAPPA were co-transfected (Figure 5C). Western blot analysis also showed that the levels of the p-STAT3 and STAT3 proteins were down-regulated by knocking down circPAPPA or overexpressing miR-384. However, this process could also be partly reversed with the co-transfection of an miR-384 inhibitor and si-circPAPPA (Figure 5D,E). The STAT3 pathway has been reported to be associated with PE, and studies have shown that STAT3 is a key regulator of trophoblast proliferation and invasion [19,20]. In addition, decreased expression and activation of STAT3 have been detected in PE patients [21]. Here, we found that the expression of STAT3 mRNA was decreased in PE placental tissues (Figure 5F). Western blot results also indicated that the expression of p-STAT3 and STAT3 proteins in PE placenta tissues were significantly lower than in the normal control group (Figure 5G,H).

## Discussion

PE is a common pregnancy complication characterized by hypertension and proteinuria; its pathogenesis remains to be elucidated. Placental trophoblast dysfunction is the main pathology of PE. It is believed that PE might be caused by inadequate placental development, trophoblast invasion, and placental ischemia [22,23].

circRNAs, a novel class of endogenous non-coding RNAs formed by a covalently closed loop, have been shown to regulate cellular functions and to be involved in the etiologies of numerous disorders [24]. Studies on PE and circRNAs are still in the preliminary stages, but in a recent report, differential circRNA expression profiles were observed in blood and placenta tissues of PE patients [11,25]. Using clinical correlation studies, some differentially expressed circRNAs, such as hsa_circ_0036877, circRNA-0004904, and circRNA-0001855, might function as competing endogenous RNAs and serve as novel blood biomarkers for the early prediction of PE [26,27]. In the present study, we evaluated the possible functions of a circRNA, circPAPPA, which we previously characterized. We found that the expression of circPAPPA was significantly decreased in placenta and plasma of PE patients. Further functional studies showed that knockdown of circPAPPA inhibited cell proliferation, and also suppressed the invasion abilities of trophoblast cells as revealed by an *in-vitro* Transwell assay. We then further characterized the potential molecular mechanism of the function of circPAPPA in PE. Based on the classical regulatory model of circRNA as a miRNA sponge, we used bioinformatics software to predict miRNAs that might interact with it. Using the luciferase reporter gene assay, we confirmed that miR-384 directly binds to circPAPPA, and showed that luciferase activity was significantly decreased by co-transfection of miR-384 mimics and the wild-type form of circPAPPA. FISH results showed that circPAPPA co-localized with miR-384 in the cytoplasm. Overall, these results suggest that circPAPPA exerts its regulatory function by sponging miR-384. miR-384 has been reported to be down-regulated in multiple human cancers, suggesting that it has a tumor-suppressive role [16,17]. However, the role of miR-384 in PE had not been investigated previously. We examined the expression of miR-384 in PE placenta tissues, and found that it was up-regulated compared with the levels in normal controls, indicating an inverse correlation with circPAPPA expression. Overexpressed miR-384 significantly inhibited the proliferation and invasion of trophoblast cells. Since miRNAs act by regulating target genes, we used bioinformatics prediction and the luciferase reporter assay, and showed that STAT3 is a direct target of miR-384. A member of the STAT family, STAT3 has been shown to play a crucial role in modulating the epithelial–mesenchymal transition during cancer metastasis [28], STAT3 activation is associated with cell growth, particularly tumor growth [29], and activated STAT3 can induce the expression of cell-cycle regulators, apoptosis inhibitors, and angiogenesis-inducible factors, which are all key factors in tumorigenesis [30–32,9]. Down-regulated expression of STAT3 and its phosphorylated form have been observed in extravillous trophoblasts, villous trophoblasts, and the entire placenta in patients with PE and in a rat PE model [21,33], indicating that STAT3 signaling is indispensable for the induction and regulation of trophoblast and tumor cell invasion. These findings suggest that STAT3 pathways play an important role in the pathogenesis of PE. Certain regulatory factors, such as RPS4Y1, HO-1, and HLA-G, have been reported to play a role in PE by affecting the expression and activation of STAT3 in trophoblast cells [20,34,35]. In the present study, we found that the STAT3 and p-STAT3 mRNA levels decreased following circPAPPA knockdown or miR-384 overexpression, and that this decrease could be reversed by co-transfection of miR-384 inhibitor and si-circPAPPA. Western blot analysis showed corresponding results. Furthermore, we found that the expression of p-STAT3 and STAT3 in PE placenta was significantly decreased, consistent with other reports.

Based on the above results, we speculate that the down-regulation of circPAPPA decreased the expression of STAT3 by sponging miR-384, and decreased expression of STAT3 protein may contribute to decreased phosphorylated STAT3 levels, which subsequently suppressed proliferation and the invasiveness of trophoblast cells by regulating related target gene transcription. In conclusion, our study reveals a preliminary function of circPAPPA in trophoblast cells. Further in-depth studies are needed to elucidate its biological functions to obtain a comprehensive understanding of its role in trophoblast cells, which are associated with PE. The present study provides new insights for further studies, and identifies a potential new biomarker for the diagnosis and treatment of PE. In addition, the results have implications in other signaling cascades that are regulated by STAT3 and related transcription factors in cancer and other inflammatory diseases.

## Abbreviations

circPAPPA: circular RNA of pregnancy-associated plasma protein A
circRNA: circular RNA
EdU: 5-ethynyl-2′-deoxyuridine
FBS: fetal bovine serum
FISH: fluorescence *in situ* hybridization
FITC: fluorescein isothiocyanate
GAPDH: glyceraldehyde 3-phosphate dehydrogenase
PAPPA: pregnancy-associated plasma protein A
PBS: phosphate-buffered saline
PE: preeclampsia
qRT-PCR: quantitative real-time polymerase chain reaction
STAT3: signal transducer and activator of transcription 3

## References

[1] Redman C.W. and Sargent I.L. (2005) Latest advances in understanding preeclampsia. Science 308, 1592 10.1126/science.1111726 15947178

[2] Roberts J.M. and Gammill H.S. (2005) Preeclampsia: recent insights. Hypertension 46, 1243–1249 10.1161/01.HYP.0000188408.49896.c5 16230510

[3] Edgardo A., Cristina C., Grosso A.L., Doris C. and Lale S. (2013) Global and regional estimates of preeclampsia and eclampsia: a systematic review. Eur. J. Obstet. Gynecol. Reprod. Biol. 170, 1–7 23746796 10.1016/j.ejogrb.2013.05.005

[4] Roberts J.M. and Escudero C. (2012) The placenta in preeclampsia. Obstet. Gynecol. Ann. 2, 72–83 10.1016/j.preghy.2012.01.001 PMC3381433 22745921

[5] Roberts J.M. and Hubel C.A. (2009) The two stage model of preeclampsia: variations on the theme. Placenta 30, 32–37 10.1016/j.placenta.2008.11.009 PMC2680383 19070896

[6] Hanan M., Soreq H. and Kadener S. (2017) CircRNAs in the brain. RNA Biol. 14, 1028–1034 10.1080/15476286.2016.1255398 27892769 PMC5680707

[7] Zhao Z., Li X., Jian D., Hao P., Rao L. and Li M. (2017) Hsa_circ_0054633 in peripheral blood can be used as a diagnostic biomarker of pre-diabetes and type 2 diabetes mellitus. Acta Diabetol. 54, 237–245 10.1007/s00592-016-0943-0 27878383 PMC5329094

[8] Zhang H.D., Jiang L.H., Sun D.W., Hou J.C. and Ji Z.L. (2018) CircRNA: a novel type of biomarker for cancer. Breast Cancer 25, 1–7 10.1007/s12282-017-0793-9 28721656

[9] Huang Z., Zhou W., Li Y., Cao M., Wang T., Ma Y. (2018) Novel hybrid molecule overcomes the limited response of solid tumours to HDAC inhibitors via suppressing JAK1-STAT3-BCL2 signalling. Theranostics 8, 4995–5011 10.7150/thno.26627 30429882 PMC6217055

[10] Li J., Liu Y.Y., Yang X.F., Shen D.F., Sun H.Z., Huang K.Q. (2018) Effects and mechanism of STAT3 silencing on the growth and apoptosis of colorectal cancer cells. Oncol. Lett. 16, 5575–5582 30344711 10.3892/ol.2018.9368 PMC6176248

[11] Qian Y., Lu Y., Rui C., Qian Y., Cai M. and Jia R. (2016) Potential significance of circular RNA in human placental tissue for patients with preeclampsia. Cell. Physiol. Biochem. 39, 1380–1390 10.1159/000447842 27606420

[12] Zhou W., Wang H., Wu X., Long W., Zheng F., Kong J. (2018) The profile analysis of circular RNAs in human placenta of preeclampsia. Exp. Biol. Med. 243, 1109–1117 10.1177/1535370218813525 PMC6327373 30458645

[13] Livak K.J. and Schmittgen T.D. (2001) Analysis of relative gene expression data using real-time quantitative PCR and the 2(-Delta Delta C(T)) Method. Methods 25, 402–408 10.1006/meth.2001.1262 11846609

[14] Peter K., Simon B. and Berthold H. (2003) Endovascular trophoblast invasion: implications for the pathogenesis of intrauterine growth retardation and preeclampsia. Biol. Reprod. 69, 1–7 10.1095/biolreprod.102.014977 12620937

[15] Pijnenborg R., Vercruysse L. and Hanssens M. (2008) Fetal-maternal conflict, trophoblast invasion, preeclampsia, and the red queen. Hypertens. Pregnancy 27, 183–196 10.1080/10641950701826711 18484423

[16] Wang F. (2019) miR-384 targets metadherin gene to suppress growth, migration, and invasion of gastric cancer cells. J. Int. Med. Res. 47, 926–935 10.1177/0300060518817171 30614349 PMC6381512

[17] Yan L., Wu K., Du F., Yin X. and Guan H. (2018) miR-384 suppressed renal cell carcinoma cell proliferation and migration through targeting RAB23. J. Cell. Biochem. 120, 1420–1426 10.1002/jcb.27180 30390327

[18] Zheng J., Liu X., Wang P., Xue Y., Ma J., Qu C. (2016) CRNDE promotes malignant progression of glioma by attenuating miR-384/PIWIL4/STAT3 Axis. Mol. Ther. 24, 1199–1215 10.1038/mt.2016.71 27058823 PMC5088760

[19] Chang X., Bian Y., He Q., Yao J., Zhu J., Wu J. (2017) Suppression of STAT3 signaling by Delta9-Tetrahydrocannabinol (THC) induces trophoblast dysfunction. Cell. Physiol. Biochem. 42, 537–550 10.1159/000477603 28578322

[20] Chen X., Tong C., Li H., Peng W., Li R., Luo X. (2018) Dysregulated expression of RPS4Y1 (Ribosomal Protein S4, Y-Linked 1) impairs STAT3 (Signal Transducer and Activator of Transcription 3) signaling to suppress trophoblast cell migration and invasion in preeclampsia. Hypertension 71, 481–490 10.1161/HYPERTENSIONAHA.117.10250 29378854

[21] Zhang Z., Yang X., Zhang L., Duan Z., Jia L., Wang P. (2015) Decreased expression and activation of Stat3 in severe preeclampsia. J. Mol. Histol. 46, 205–219 10.1007/s10735-015-9613-8 25752465

[22] Philippe M., Lionel C., Jean-Claude C., Michèle R., Michel B. and Serge U. (2004) Pathophysiology of preeclampsia: links with implantation disorders. Eur. J. Obstet. Gynecol. Reprod. Biol. 115, 134–147 10.1016/j.ejogrb.2003.12.030 15262345

[23] Redman C.W. and Staff A.C. (2015) Preeclampsia, biomarkers, syncytiotrophoblast stress, and placental capacity. Am. J. Obstet. Gynecol. 213, S9.e1,S9–S11, 10.1016/j.ajog.2015.08.003 26428507

[24] Memczak S., Jens M., Elefsinioti A., Torti F., Krueger J., Rybak A. (2013) Circular RNAs are a large class of animal RNAs with regulatory potency. Nature 495, 333–338 10.1038/nature11928 23446348

[25] Zhang Y.G., Yang H.L., Long Y. and Li W.L. (2016) Circular RNA in blood corpuscles combined with plasma protein factor for early prediction of pre-eclampsia. BJOG 123, 2113–2118 10.1111/1471-0528.13897 26846540

[26] Hu X., Ao J., Li X., Zhang H., Wu J. and Cheng W. (2018) Competing endogenous RNA expression profiling in pre-eclampsia identifies hsa_circ_0036877 as a potential novel blood biomarker for early pre-eclampsia. Clin. Epigenet. 10, 48 10.1186/s13148-018-0482-3 29643944 PMC5891938

[27] Jiang M., Lash G.E., Zhao X., Long Y., Guo C. and Yang H. (2018) CircRNA-0004904, CircRNA-0001855, and PAPP-A: potential novel biomarkers for the prediction of preeclampsia. Cell. Physiol. Biochem. 46, 2576–2586 10.1159/000489685 29758559

[28] Corvinus F.M., Fitzgerald J.S., Friedrich K. and Markert U.R. (2003) Evidence for a correlation between trophoblast invasiveness and STAT3 activity. Am. J. Reprod. Immunol. 50, 316–321 10.1034/j.1600-0897.2003.00099.x 14672334

[29] Jiang G.X., Zhong X.Y., Cui Y.F., Liu W., Tai S., Wang Z.D. (2010) IL-6/STAT3/TFF3 signaling regulates human biliary epithelial cell migration and wound healing in vitro. Mol. Biol. Rep. 37, 3813–3818 10.1007/s11033-010-0036-z 20229017

[30] Couto J.P., Daly L., Almeida A., Knauf J.A., Fagin J.A., Sobrinho-Simoes M. (2012) STAT3 negatively regulates thyroid tumorigenesis. Proc. Natl. Acad. Sci. U.S.A. 109, E2361–2370 10.1073/pnas.1201232109 22891351 PMC3435219

[31] Chun J., Li R.J., Cheng M.S. and Kim Y.S. (2015) Alantolactone selectively suppresses STAT3 activation and exhibits potent anticancer activity in MDA-MB-231 cells. Cancer Lett. 357, 393–403 10.1016/j.canlet.2014.11.049 25434800

[32] Bo C., Wu Q., Zhao H., Li X. and Zhou Q. (2018) Thymosin alpha1 suppresses migration and invasion of PD-L1 high-expressing non-small-cell lung cancer cells via inhibition of STAT3-MMP2 signaling. Onco Targets Ther. 11, 7255–7270 10.2147/OTT.S177943 30425517 PMC6205819

[33] Zhang Z., Wang X., Wang J. and Zhang L. (2018) The decreased expression of Stat3 and p-Stat3 in preeclampsia-like rat placenta. J. Mol. Histol. 49, 175–183 10.1007/s10735-018-9757-4 29353343

[34] Liu X., Gu W. and Li X. (2013) HLA-G regulates the invasive properties of JEG-3 choriocarcinoma cells by controlling STAT3 activation. Placenta 34, 1044–1052 10.1016/j.placenta.2013.07.070 24054889

[35] Qu H.M., Qu L.P., Li X.Y. and Pan X.Z. (2018) Overexpressed HO-1 is associated with reduced STAT3 activation in preeclampsia placenta and inhibits STAT3 phosphorylation in placental JEG-3 cells under hypoxia. Arch. Med. Sci. 14, 597–607 10.5114/aoms.2016.63261 29765448 PMC5949914

